# Problems in Postmortem Pathology Training

**DOI:** 10.5146/tjpath.2022.01569

**Published:** 2023-01-15

**Authors:** Ali Rıza Tümer, Emirhan Eskicioğlu, Cenk Sökmensüer, Tuğçe Fındıkoğlu

**Affiliations:** Department of Forensic Medicine, Hacettepe University Faculty of Medicine, Ankara, Turkey; Department of Pathology, Hacettepe University Faculty of Medicine, Ankara, Turkey

**Keywords:** Forensic pathology, Forensic medicine, Medical residency

## Abstract

***Objective:*** In Turkey, autopsy performers, namely forensic medicine practitioners, are neither pathologists nor have properly received pathology training during residency in contrast to the Anglo-Saxon model of forensic medicine practices, since the current curriculum of forensic medicine residency lacks adequate training in post-mortem histopathology. Likewise, pathologists lack a specific post-mortem pathology clerkship. In this study, we intended to determine whether forensic physicians in Turkey find themselves competent in post-mortem histopathology or were adequately trained during their residencies.

***Material and Method:*** Turkish forensic medicine practitioners were administered an online questionnaire whereby self-evaluations of their histopathology knowledge and their views on histopathology training during forensic medicine residency were assessed. The 151 physicians who completed the questionnaire made up the study group.

***Results:*** It was found out that the majority of Turkish forensic medicine practitioners (85.4%) did not find the histopathology training during their residency adequate. Similarly, 85.4% of the participants indicated their incompetence in histopathological examination of post-mortem tissue of any kind, and showed their willingness for further training in pathology. 66.9% strongly agreed that post-mortem histopathology requires training that is distinct from surgical pathology. In case of providing post-mortem histopathology training within the scope of forensic medicine residency, topics such as microscopic morphology of post-mortem changes, histological changes related to injuries, and estimation of wound age are expected to be beneficial to 88.7% 83.4%, and 83.4% of the participants respectively.

***Conclusion:*** The current curriculum should be revised in a way that the surgical pathology clerkship meets forensic physicians’ needs, so that they can then refer more difficult, non-routine histopathological consultations to pathologists who are also well-trained in postmortem histopathology. Consideration should also be given to establishing a subspecialty training - a master’s or doctoral degree programs in forensic pathology.

## INTRODUCTION

Postmortem histopathology is a field of medical sciences that examines tissues retrieved from a deceased individual under a light microscope for various purposes (1). In contrast to the Anglo-Saxon model of forensic medicine practices, forensic physicians’ interest and competence in the field of histopathology varies in the Middle East and Continental European countries, where forensic medicine practitioners are separate from physicians who perform post-mortem histopathological examinations. There is a similar situation in Turkey, as the forensic medicine structure was adapted from Continental Europe.

Postmortem histopathology deals with the forensic evaluation of a post-mortem tissue in any context, and the significance of a particular finding as forensic evidence. Post-mortem histopathology includes basic phenomena such as post-mortem changes in cells and tissues, evidence of vitality at the time a lesion occurred, the mechanism of injuries, and the ruling out or confirmation of any pathological changes in identifying the cause of death (2). Histopathology is a routine part of post-mortem examination and can provide useful information for case solution, not only in cases where the cause of death cannot be determined by macroscopic findings, but even when the cause of death is apparent to the naked eye at autopsy (3).

In post-mortem examinations around the world, generally speaking, there are two types of forensic autopsy performers: pathologists who are subspecialized in forensics and physicians who undergo forensic medicine residency training. In Turkey, autopsy performers are not pathologists and pathologists do not regularly perform autopsy (forensic or medical) and lack structured training of post-mortem histopathology.

In this study, we surveyed forensic physicians in Turkey to determine whether they were trained in the field of post-mortem histopathology during their residency, whether they had ever performed a histopathological examination of a post-mortem specimen, if they have access to basic histopathology equipment, and if any revision should be applied to forensic medicine residency program so that a collaborative and goal-oriented training strategy with pathologists is established.

## MATERIALS and METHODS

As the study population, it was intended to include all physicians actively practicing forensic medicine in Turkey. The number is known to be around 800 according to the data provided by the Association of Forensic Medicine Specialists (ATUD). Invitations to participate were made by sending out the online questionnaire via a platform accessible to all Turkish forensic medicine practitioners. Online notifiers were issued at two-week intervals for 4 times and the invites must have reached 427 forensic medicine practitioners considering that the platform has that quantity of members. Eventually a total of 151 physicians (response rate: 151/427, 35.4%) who completed the questionnaire made up the study group.

The online survey method was chosen as the method of data collection. In terms of structure, the questionnaire consists of four sets of questions. The first set aims to determine the academic titles of the participants, the institutions they work in, and their access to a light microscope. The second set of questions concerns the study group’s self-evaluation of their histopathological competency. Their views on the formation of histopathology as well as the topics expected to be beneficial in histopathology training during forensic medicine residency training were asked with the third set of questions, and finally, the fourth set determined their preference of forensic histopathological practice.

This study was reviewed and approved by the Non-Interventional Clinical Research Ethics Committee of Hacettepe University (issue date 02/03/2021, number 2021/05-31).

## RESULTS

Table 1 presents the academic titles of the participants and the institutions where they completed their residency. Among all participants, residents composed more than half (55.0%, n=83) while the rest were attending physicians (27.8%, n=42) and professors (17.2%, n=26). In Turkey, forensic medicine residency is available in three structurally different institutions: training and research hospitals, the Council of Forensic Medicine (under the Ministry of Justice), and universities. Most of the participants (80.8%, n=122) were currently residents or had completed their residency at a university.

**Table 1:** Participants’ academic titles and institutions where their residency took place.

	**Institution where their residency took place**			**Total**
	**Faculty of Medicine (Universities)**	**The Council of Forensic Medicine (Ministry of Justice)**	**Training and Research Hospital (Ministry of Health)**	
**Academic Title**	**n (%)**	**n (%)**	**n (%)**	**n (%)**
Resident/Research Assistant	71 (85.5)	2 (2.4)	10 (12.0)	83 (55.0)
Attending Physician/Specialist	34 (81.0)	8 (19.0)	0 (0.0)	42 (27.8)
Professor	17 (65.4)	9 (34.6)	0 (0.0)	26 (17.2)
**Total**	122 (80.8)	19 (12.6)	10 (6.6)	151 (100.0)

When asked about the availability of a light microscope, 92 physicians (60.9%) stated that they do not have a light microscope available at work while 42 (27.8%) stated that they can use a light microscope at will. The use of microscope was stated to be subject to some permissions for 17 (11.3%) of 151 participants.

The participants’ self-evaluations of the histopathology training during their residency are presented in Table 2. The survey revealed the majority (85.4%, n=129) do not find histopathology training during their residency to be adequate. Similarly, 85.4% (n=129) of participants indicate their incompetence in histopathological examination of post-mortem tissue of any kind, with only a small portion (2.0%, n=3) claiming competence in post-mortem histopathological examination.

**Table 2:** The participants’ self-evaluations of their histopathology training.

	**Do you find the histopathology training during your residency adequate?**		**Do you consider yourself competent in examining any postmortem tissue under the light microscope?**			**Total**
	**No**	**Yes**	**No**	**Slightly, yes**	**Yes**	
**Academic Title**	**n (%)**	**n (%)**	**n (%)**	**n (%)**	**n (%)**	**n (%)**
Resident/Research Assistant	73 (88.0)	10 (12.0 )	74 (89.2 )	8 (9.6)	1 (1.2)	83 (100.0)
Attending Physician/Specialist	22 (84.6)	4 (15.4)	19 (73.1)	6 (23.1)	1 (3.8)	26 (100.0)
Professor	34 (81.0)	8 (19.0)	36 (85.7)	5 (11.9)	1 (2.4)	42 (100.0)
**Total**	129 (85.4)	22 (14.6)	129 (85.4)	19 (12.6)	3 (2.0)	151 (100.0)

Using a five-point Likert-type scaling, the views of participants on the formation of histopathology training were determined and the data are shown in Table 3. 66.9% (n=101) strongly agree to the proposal that post-mortem histopathology requires a distinct training from conventional pathology, while 1.3% (n=2) strongly disagree and 3.3% (n=5) are indecisive. Among 151 participants, 94 (62.3%) strongly and 42 (27.8%) moderately agree that additional post-mortem histopathology training should be available throughout forensic medicine residency. 61 subjects (40.4%) strongly and 56 (37.1%) moderately agree that performing a certain number of histopathologic examinations should be obligatory in order to complete forensic medicine residency, while 12 (7.9%) slightly and 12 (7.9%) totally disagree. Among physicians enrolled in this study, 63 of 151 (41.7%) strongly disapprove the statement that there are adequate research and studies in the field of post-mortem histopathology. While 49 subjects (32.5%) remained undecided, 8 (5.3%) slightly approved. It was revealed that no participant strongly agreed with that statement.

**Table 3:** Views of participants on the formation of histopathology training.

	**Strongly agree**	**Slightly agree**	**Indecisive**	**Slightly disagree**	**Strongly disagree**
	**n (%)**	**n (%)**	**n (%)**	**n (%)**	**n (%)**
Do you agree that postmortem histopathology requires a different training than surgical pathology?	101 (66.9)	41 (27.2)	5 (3.3)	2 (1.3)	2 (1.3)
Do you agree that postmortem histopathology training should be available within the scope of forensic medicine residency?	94 (62.3)	42 (27.8)	7 (4.6)	4 (2.6)	4 (2.6)
Should there be an obligation to perform a certain number of histopathological examinations throughout forensic medicine residency?	61 (40.4)	56 (37.1)	10 (6.6)	12 (7.9)	12 (7.9)
Do you believe that the number of studies and the research in the field of post-mortem histopathology are adequate in Turkey?	0 (0.0)	8 (5.3)	49 (32.5)	31 (20.5)	63 (41.7)

In case of providing post-mortem histopathology training within the scope of forensic medicine residency, topics such as microscopic morphology of post-mortem changes, histological changes related to injuries, estimation of wound age, vitality markers of wound, and tissue handling (sampling, transportation and preservation) during autopsy are expected to be beneficial to 88.7% (n=134), 83.4% (n=126), 83.4% (n=126), 81.5% (n=123), and 79.5% (n=120) of the participants respectively (Table 4).

**Table 4:** Topics expected to be beneficial in case of providing post-mortem histopathology training.

	**n (%)**		**n (%)**		**n (%)**
Microscopic morphology of post-mortem changes	134 (88.7)	Basic histological staining	101 (66.9)	Immunohistochemical methods	80 (53.0)
Estimation of wound age	126 (83.4)	Histological changes related to intoxication	100 (66.2)	Microscopic morphology of common infectious diseases	76 (50.3)
Histological changes related to injury	126 (83.4)	Thromboembolism	100 (66.2)	Biological identification with pathology	66 (43.7)
Vitality markers of wound	123 (81.5)	Histological changes related to thermal injury	95 (62.9)	Neuro-histopathology	59 (39.0)
Tissue handling during autopsy (sampling, transportation and preservation)	120 (79.5)	Fetal-Perinatal-Newborn Histopathology	94 (62.2)	Basic training enough to distinguish dysplasia	1 (0.7)
Cardiac histopathology	116 (76.8)	Intro to general histology, microscopic morphology of various tissues	94 (62.2)	Basic pathological changes such as blood extravasation, edema, inflammation, tumor, necrosis	1 (0.7)
Histological changes related to asphyxia	107 (70.9)	Histological artifacts	92 (60.9)	Sperm detection in sexual assaults	1 (0.7)

At the end of the questionnaire, two distinct practices of whether post-mortem histopathological examination should be carried out by the medical examiner (who is not essentially a pathologist) who performs the autopsy or by a different pathology specialist were presented. Roughly half (50.3%, n=77) of the participants indicated professionals who perform autopsy and post-mortem histopathological tissue examination should be one and the same, as in the West (in the UK and the USA), while 49.7% (n=74) supported the idea that the medical examiner performing autopsy should consult someone else and especially a forensic pathologist when needed, as in some European countries like Germany, Greece, and Italy.

## DISCUSSION

In addition to the external examination of the deceased and autopsy, histopathological study is carried out as an integral part of post-mortem investigations (4). Post-mortem histopathological examination aiming to verify abnormal findings discovered at autopsy and sometimes to guide medicolegal investigations (2), has different practices in various forensic science schools. This distinction is primarily apparent between physicians who perform forensic autopsies and the ones carrying out histopathological examination.

In the USA and the UK, post-mortem histopathological specimens are evaluated by the same specialists who perform autopsies if a microscopic assessment is required. Professionals performing autopsy in the USA and the UK specialize in pathology. Within a subspecialty program called “forensic pathology”, those pathology specialists are trained in various fields of forensic sciences such as anthropology, toxicology, wound ballistics, and blood and body fluid analysis, and consequently become forensic pathology specialists (5,6). In other words, those physicians become “forensic medicine specialists” after undergoing a pathology specialization training.

In European countries such as Germany, Greece, and Italy, tissue specimens obtained at autopsy are examined under the light microscope and reported by distinct physicians specialized in the field of histopathology.

It is seen that physicians performing autopsies begin their forensic medicine residency directly after graduation from the faculty of medicine and undergo histopathology training throughout their post-graduate specialization training. This clinical clerkship in histopathology is usually time-limited and takes place in pathology laboratories. Being compulsory for 6 months and elective for 6 more, those rotations could be provided for a total of 12 months in Germany (7), whereas in France this clerkship is available as an elective anatomic pathology training for 2 semesters of total 8 academic-term-long forensic medicine residency program (8). The medical literature lacks publications about the competence of European forensic medicine practitioners in post-mortem histopathological examination except a few papers mentioning that in Germany, “forensic medicine” is regarded as a distinct medical specialty called “Rechtsmedizin” whilst histopathology is an integral element of residency training, and to examine a total of 2,000 (two thousand) histological specimens is a must in order to complete the specialization program (9).

Autopsies in Turkey are carried out by physicians, who have completed a four-year residency program in forensic medicine that does not include extensive training in histopathology, and tissue specimens obtained during autopsies are then referred to pathologists after being appropriately collected for microscopic assessment.

Despite not being a member state of the European Union, Turkish medical education authorities adapted the European system and structured the forensic medical residency training so that residents would experience a two-month-long histopathology clerkship in surgical pathology, whose goals are to earn competence in the macroscopic sampling and the interpretation of pathology reports as outlined in the “Core Curriculum for Residency in Forensic Medicine v2.4. (2018-2019)” established by Board of Specialization in Medicine (under the Ministry of Health) (10). Hence it is deductible that actively performing the microscopic analysis of the tissue directly is not among the competence goals of the curriculum.

We intend to draw attention to these two distinct practices of post-mortem examinations by trying to find out whether this long-standing tradition in Turkish forensic medicine practices exists due to inadequacy of histopathology training during the forensic medicine residency program,

Considering that earning a “professor” title takes at least 15 years in the profession and that 81.0% of the professors declared inadequate histopathology training (Table 2), the forementioned lack of proper pathology training in forensic medicine residency program must have existed even at those times that they were residents, because apart from personal interest, microscopic examination does not fall within the everyday practice routine of a forensic medicine physician.

Pathologists deal mostly with biopsies and intraoperatively resected tissues/organs during their training, though, at their discretion, they may have a chance to work for a very limited time in laboratories that examine post-mortem tissues. Even when they are required to perform post-mortem examinations (which is also seldom), these are mostly medical autopsies of fetus and newborns. Thus, the only way a pathologist completing residency in Turkey could undergo post-mortem histopathology training is by working at a laboratory or a clinic where post-mortem investigations are constantly performed, although pathology residents are obliged to perform autopsies in order to complete their residencies. Besides, post-mortem biopsy is another procedure that can be carried out if given approval.

In addition, there is no clerkship defined in the “Core Curriculum for Residency in Surgical Pathology v2.4 (2019)” for surgical pathology (11). In fact, apart from cytopathology, there is no officially defined subspecialty of surgical pathology in Turkey (12).

This state of inadequacy also hinders the forensic medicine residents in surgical pathology clerkships from receiving satisfying training on the examination of post-mortem tissues as it would not be legitimate to expect a pathologist lacking sufficient experience in post-mortem examination to instruct or guide another physician on this issue.

From the perspective of forensic medicine physicians, it was agreed by 142 of the total of 151 (94.0%) participants that post-mortem histopathology training requires a different training than conventional surgical pathology (Table 3). In order to acquire some competency in microscopic examinations, 117 participants (77.5%) supported an obligation to perform a certain number of histopathological examinations throughout forensic medicine residency as in the German system.

As they demand forensics-oriented training, some specific topics are expected to be advantageous if learnt (Table 4): microscopic morphology of post-mortem changes (88.7%), estimation of wound age (83.4%), histological changes related to injury (81.5%), and histological changes related to asphyxia (70.9%). Even though some pathology-specific topics like neuro-histopathology (39.0%) or immunohistochemical methods (53.0%) are revealed to be less demanded by forensic physicians, getting to know cardiac histopathology appears to be beneficial to the majority of forensic physicians (76.8%). The most likely reason is that heart dissection takes place in each autopsy performance.

## CONCLUSIONS

Regardless of which system is adopted, the Anglo-Saxon or the European one, post-mortem histopathological investigations are executed by pathology specialists, while autopsies could be performed by both pathologists and non-pathologist forensic medicine physicians.

In spite of the fact that Turkish forensic medicine practitioners are willing to acquire some microscopy skills, lack of pathologists trained in forensic sciences constitutes a barrier to provide adequate training. In Turkey, most of the autopsies are executed in divisional branches of the Council of Forensic Medicine. Therefore, the pathologists at those clinics gain experience in post-mortem investigations by examining tissue specimens obtained during autopsy on a regular basis. However, in other institutions where forensic autopsies do not take place, the pathologists would not have the opportunity to examine or be acquainted with post-mortem tissue specimens, and thus would lack adequate knowledge to instruct the residents of forensic medicine during their pathology clerkships.

Within the frame of the current forensic medicine residency curriculum, it would be almost impossible to gain adequate experience and knowledge in microscopic examinations since a 2-month-long clerkship would not be enough to get acquainted with a sufficient number of specimens. Considering the practices around the world, at least one year of pathology training specifically focused on post-mortem microscopic morphology should be provided for forensic medicine residents. Similarly, at least 3-month-long post-mortem histopathology clerkships should be established for pathology residents in institutions where forensic autopsy is a routine procedure.

Since the current practice of autopsies being performed by forensic medicine physicians and histopathological examinations being carried out by pathologists stays on track, there is no need for a forensic medicine practitioner to take over the role of pathologist. Nonetheless, in order to meet international standards in forensic medicine residency training, the authors feel that the current curriculum should be revised in a way that the surgical pathology clerkship meets forensic physicians’ needs and that the competence goals include being able to identify basic and most common pathological phenomena under the light microscope. Forensic medicine physicians can then refer more difficult, non-routine histopathological consultations to pathologists who are also well-trained in postmortem histopathology.

We hope this study will encourage both forensic medicine and pathology physicians to review their residency trainings, so that forensic medicine practitioners could be provided with goal-oriented pathology clerkships rotations to gain experience in basic histopathological examinations. Consideration should also be given to establishing a subspecialty training - a master’s or doctoral degree programs in forensic pathology - and made available to forensic medicine, histopathology and anatomy practitioners if the above mentioned suggestions to the current curricula appear to be impractical at the moment. Even further, how forensic medicine and pathology specialists could collaborate to perform forensic autopsies and how such settings could be ensured are other questions of importance.

## Conflict of Interest

The authors declare no conflict of interest.
